# A Numerical Study on the Mesoscopic Characteristics of Ti-6Al-4V by Selective Laser Melting

**DOI:** 10.3390/ma15082850

**Published:** 2022-04-13

**Authors:** Xiaohui Ao, Jianhua Liu, Huanxiong Xia, Ye Yang

**Affiliations:** 1 School of Mechanical Engineering, Beijing Institute of Technology, Beijing 100081, China; xhao@bit.edu.cn (X.A.); jeffliu@bit.edu.cn (J.L.); 2 Yangtze Delta Region Academy of Beijing Institute of Technology, Jiaxing 314000, China; 3 School of Mechanical and Material Engineering, North China University of Technology, Beijing 100144, China; yangye@ncut.edu.cn

**Keywords:** selective laser melting, additive manufacturing, multiphase flow, molten pool

## Abstract

Selective laser melting is a typical powder-bed additive manufacturing technology, for which it is difficult and expensive to observe and measure the molten pool due to its short lifetime and tiny size. This paper introduced a two-stage mesoscopic layer-by-layer simulation framework for the numerical study of the SLM process, where the powder laying and laser scanning are included and conducted alternatively. For the simulation of powder laying, the dynamic behaviors of the particles as well as the particle–particle and particle–scraper interactions are included. For the simulation of laser scanning, a coupled multi-phase and multi-physics system was considered, where the effects of surface tension, Marangoni effect, and vapor recoil are considered, and the behaviors of heat transfer, fluid flow, and melting/solidification are simulated. This simulation framework was then used to simulate the Ti-6Al-4V SLM process. The evolutions of the molten pool and track were presented, and the characteristics of the molten pool, keyhole, and track were analyzed and discussed, specifically, the effects of the laser power and scanning speed on the three-dimensional morphology and size of the molten pool were numerically studied, and their dependencies were discussed and found.

## 1. Introduction

Selective Laser Melting (SLM) is an additive manufacturing technology, which has a wide range of application prospects in aerospace, automobile manufacturing, medical equipment, and other fields [1,2,3]. The fundamental principle of the SLM process is to melt the powder bed selectively with a laser and then form a part after cooling and solidification layer by layer. Defects that may appear in the process include pores, spheroidization, cracks, etc. [4], which will weaken the mechanical performance of the part. To clarify the formation mechanism of the aforementioned defects, it is necessary to analyze the dynamic process of the SLM process. However, since the size of the molten pool in the SLM process is usually hundreds of microns and the melting and solidification of the powder occur in hundreds of microseconds, it is costly to conduct dynamic observations through experiments. Although researchers have performed real-time dynamic observations of the SLM process [5,6,7,8] and captured the molten pool image and the movement of powders and melt, this solution is expensive and cannot provide information on the temperature evolution. Additionally, the SLM process contains a variety of complex physical phenomena, such as melt flow, Marangoni effect, evaporation, phase change, thermal radiation, etc. [9], and the dynamic behaviors of the molten pool, and the evolution of the temperature field significantly affect the forming defects and microstructure [10,11]. Therefore, developing a mesoscale model including heat transfer, fluid flow, and melting/solidification for the molten pool is of great significance for understanding and optimizing the SLM process.

DEM (Discrete Element Method) and CFD (Computational Fluid Dynamics) methods are popularly applied to simulate the powder laying and laser scanning of the SLM process, respectively. Chen et al. [12,13] presented a detailed numerical study on the powder-laying process by carrying out high-quality DEM simulations, which gave significant insight into the powder dynamics. Gu et al. [14,15,16] used the Finite Volume Method (FVM) to simulate the behavior of the molten pool of the SLM process and pointed out that the Marangoni effect, laser power, and sweep speed greatly affect the temperature field evolution, size of the molten pool, and the liquid phase lifetime, in which the Marangoni effect intensifies the convective heat transfer, increasing the width of the molten pool and decreasing the depth. They found that the scanning speed influences the width and surface topography of the single track more than the laser power. They also claimed that either too low or too high laser power will lead to a poor surface quality of the solidified track, specifically, low power could result in insufficient melting while high power could result in a “self-spheroidization” effect due to excessive melting. Khairallah et al. [17,18] used an Arbitrary Lagrangian Euler method to simulate the formation mechanism of pores, sputtering, and denudation zones and found that a thicker layer could result in narrow and deep depressions. Tang et al. [19] combined the DEM and CFD to simulate the SLM process with a single track and found that the surface roughness of the previously solidified layer is a key factor affecting the local thickness of the next powder layer, which is crucial to the evolution of the molten pool. A similar simulation strategy was applied by Yuan et al. [20] to numerically study the effect of laser speed on the state of the molten pool. Yan et al. [21,22,23] developed a CFD-DEM coupling model for the selective electron beam melting process (similar to the SLM process) to study the mechanism of the formation of defects between the adjacent tracks and layers, claiming that a large-spacing and slit-aligning scanning strategy can help reduce hole defects while changing the laser sweep angle is relatively easy to cause holes. Cao [24,25] compared the compactness of the powder bed obtained from powders with different particle size distributions and layer thicknesses through DEM computations and simulated the laser scanning with one or more tracks of the SLM process by CFD. This study claimed that powders with a small diameter were conducive to obtaining a denser powder bed, which in turn facilitates the forming of parts, and the formation of gasification resulted from too large volumetric energy density. Bayat et al. [26,27] introduced a heat source loading strategy considering the angle of laser incidence in the CFD model and simulated the gas-involved holes due to the keyhole and found the threshold of the keyhole’s emergence. The aforementioned DEM-CFD combined method can successfully simulate the SLM process layer-by-layer by computing the powder-laying and laser-scanning stages alternatively, in which the laser energy input is modeled by a Gaussian-body heat source model [28,29] or Gaussian-surface model [26,27].

A few simulation studies and many experimental investigations have been carried out on the SLM process with Ti-6Al-4V powder, and some preliminary fundamental aspects were reported. Zhang et al. [30] simulated the flow behavior of the molten pool of the Ti-6Al-4V SLM process using a multiphysics finite element model; however, the powders were not really shown. Jin carried out a two-dimensional numerical simulation of the Ti-6Al-4V SLM process in the powder scale using a phase-field with a finite-element method [31] and followed up with a three-dimensional simulation using a thermal fluid flow model [32], in which the effect of the laser input on the macrostructure of the molten pool was preliminary understood. The experimental study on the SLM Ti-6Al-4V currently goes much deeper than the simulation study, especially the macrostructure and microstructure of the solidified tracks, the mechanical properties of the finished part, and the effects of the process parameters on them [33,34,35,36].

This work will briefly introduce a three-dimensional mesoscopic scale simulation framework for the SLM process, realizing the alternate simulation of the powder-laying and laser-scanning processes, and then focus on the application of this framework to the SLM process using Ti-6Al-4V powder and understanding of the fundamental behaviors of heat transfer, melt flow, and solidification at the mesoscale. The three-dimensional morphology of molten pools, keyholes, and solidified tracks are numerically studied, and the response of the dimensions of the molten pool to the laser power and scanning speed is obtained. This study will help to further quantitatively understand the mesoscopic behaviors of the SLM process using Ti-6Al-4V powder and provide more data for the molten pool and its parameterized model usually used in the part-scale simulation.

## 2. Modeling and Simulation Framework

The SLM process manufactures designed parts in a manner of layer by layer, where the processing of each layer can be decomposed into two steps: powder laying and laser scanning. For the simulation of the two steps, a particle dynamics model based on the discrete element method and a multi-phase and multi-physics coupling model based on the VOF method are developed, respectively.

### 2.1. Discrete-Element Modeling of the Powder-Laying Process

In the powder-laying process, the effect of the gas on the movement of the powder particles is slight and can be ignored, and the mechanical interactions between the powders and solids, such as powder–powder, powder–scraper, and powder–wall, are moderate. These allow us to model the interactions using the theory of small deformation and elastic-damping collision in the DEM method. Please see [37] for its details.

According to Newton’s law and the angular momentum theorem, the governing equations for the momentum and angular momentum of the powder particles are given by:

(1) $$m_{i}\frac{dv_{i}}{dt}=m_{i}g+\sum_{j}\left(F_{n,ij}+F_{t,ij}\right)$$

(2) $$I_{i}\frac{d\omega_{i}}{dt}=\sum_{j}\left(R_{i}\times F_{t,ij}-\mu_{r}r_{i}F_{t,ij}\frac{\omega_{i}}{\left|\omega_{i}\right|}\right)$$

where *m_i_*, **g**, **I***_i_*, **v***_i_*, and **ω***_i_* represent the mass, gravitational acceleration, moment of inertia, translational velocity, and angular velocity of the *i*-th powder, respectively; **F**_n,*ij*_, **F**_t,*ij*_ are the normal and tangential forces between the *i*-th and *j*-th powders, respectively; *R_i_* is the vector pointing from the center of the *i*-th powder to the contact point, and its mold is the powder radius *r_i_*; *μ_r_* is the rotational friction coefficient.

### 2.2. Multi-Phase and Multi-Physics Coupling Modeling of the Laser-Scanning Process

The system is regarded as a gas–liquid two-phase system with the metal phase and the gas phase. The VOF method is used to capture the free interface. The solidifying/melting and solidified/molten metals are indicated by the solidus and liquidus temperatures, and the dynamic behavior of the solidifying/melting metal is described by using a melting-solidification model.

The characteristic velocity of fluid flow in the SLM process is about 1–10 m/s, and thus the Mach number is very low, as a consequence, the gas–metal system can be regarded as an incompressible fluid flow. Although the content of the gas could have some influence on the behavior of the molten pool. In the presented simulations, we considered the gas to be inert. That could be a rough approximation to those SLM processes protected by inert gases and even under a vacuum environment. Consequently, the mass conservation equation of the fluids can be given as

(3) ∇⋅u=0

where **u** is the velocity field. The governing equation for momentum conservation is

(4) $$\frac{\partial \rho u}{\partial t}+\nabla \cdot (\rho uu)=-\nabla p+\rho g+\nabla \cdot \mu \left(\nabla u+\nabla^{T}u\right)+S_{M}$$

where *ρ*, *p*, *μ*, **S**_M_ are the density, pressure, viscosity, and momentum source terms, respectively. Here, the momentum source terms added to this model include surface tension source term (SME), vapor recoil force source term (SMP), and melting-solidification momentum sink (SMS); please see our previous work [10] for the details of these source terms. The energy conservation equation includes the enthalpy change of the metal powder and is given as

(5) $$\frac{\partial \rho H}{\partial t}+\nabla \cdot (\rho uH)=\nabla \cdot (k\nabla T)+S_{T}$$

where *H*, *k*, *T*, *S*_T_ are the enthalpy, thermal conductivity, temperature, and energy source terms, respectively. Here, the energy source items include laser input (*S*_TL_) and heat radiation (*S*_TR_). Please also see our previous work [10] for the details.

### 2.3. Simulation Framework

**Figure 1:**
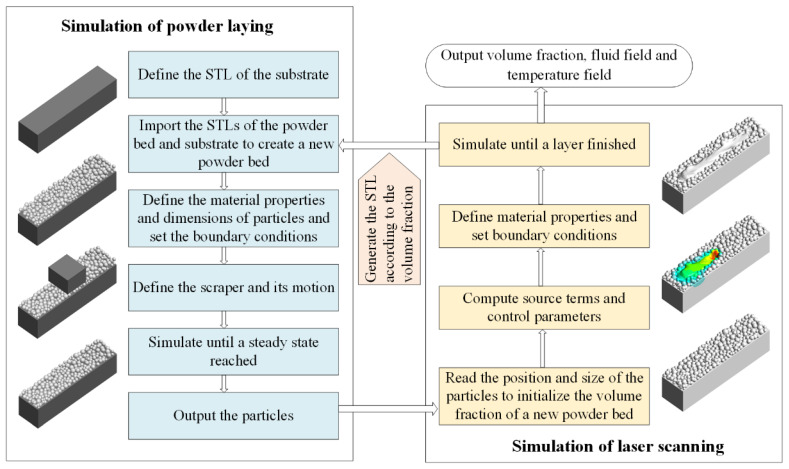
The flowchart for the simulation of SLM process.

The simulation framework is shown in Figure 1, in which the PFC3D software and the ANSYS Fluent software are used alternatively to compute the DEM model for the powder laying process and the CFD model for the laser scanning process, respectively. During the simulation of the laser-scanning process, Fluent is assisted by some user-defined functions (UDF), which are designed for the momentum and energy source terms and the loading strategy of the laser energy.

The powder-laying simulation: read the STL files of the powder bed and the scraper to generate their solid surfaces; define the movement of the scraper and specify its path and height (layer thickness of 50 μm). The powder is Ti-6Al-4V spherical particles, generated by a truncated normal distribution with a mean value of 30 μm and minimum and maximum sizes of 10 μm and 50 μm, respectively. Output the coordinates of the powders until the powder bed is stable.

The laser-scanning simulation: read the powder coordinates obtained by the powder-laying simulation to initialize the volume fraction field, and set the material properties of the fluids (the properties of the used material Ti-6Al-4V is shown in Table 1) and the boundary conditions; set the energy and momentum source items and the simulation control parameters in the UDFs, such as laser scanning speed, trajectory data, and compute the loading strategy of the heat source; generate a STL file from the volume fraction field until the scanning is completed and the melts are fully cooled down; transfer the STL file to the powder-laying simulation to generate the initial geometry for next layer.

The powder-laying simulation and laser-scanning simulation are conducted alternately until the preset trajectory data is finished.

## 3. Results and Discussions

In this section, the aforementioned simulation framework is first applied to simulate the SLM process using Ti-6Al-4V powder with single and double layers to find the fundamental mesoscopic characteristics of the molten pool, and then, a series of simulations are carried out to investigate the effect of the laser speed and power on the three-dimensional morphology evolution of the molten pool.

### 3.1. Application to the Objects with Single and Double Layers

An object with a single layer is simulated first and then followed by another layer on its top. The computational domain size is 864 × 210 × 300 (the single layer)/330 (the double layers) μm^3^, and the simulations are computed on a uniform resolution with the grid size of 3 × 3 × 3 μm^3^ and by using a fixed time step 40 ns.

**Figure 2:**
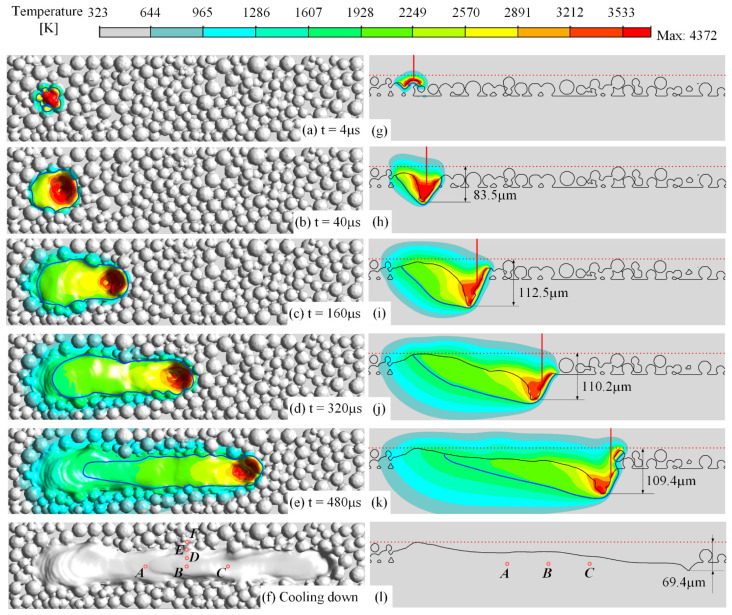
The laser-scanning process on the first layer, where the laser power is 200 W, absorptivity coefficient 0.4, scanning speed 1 m/s, and layer thickness 50 μm: (**a**–**e**) the morphology of the track and the temperature field on its surface; (**g**–**k**) the same on the longitudinal section of the powder bed, in which the red vertical line represents the central line of the laser beam, the red dashed line represents the top tangential surface of the initial powder bed, and the blue line represents the boundary of the molten pool; (**f**,**l**) the morphology of the solidified track and the same on the longitudinal section, in which 6 points (A–F) are marked to record the temperature history.

Figure 2 shows a few frames of the morphology and temperature field of the powder bed as the laser is scanning on the first layer. The laser moves from left to right, where the starting point is 100 μm to the left end of the domain, and the scanning distance is 664 μm. After the laser is turned off, the simulation does not stop until the molten pool is completely cooled down and a solidified track is obtained. At the beginning of 4 μs when the laser is irradiating the powder bed, the top surface of the powder begins to melt, as shown in Figure 2a,g, forming an initial molten pool. The temperature of the molten pool rapidly rises above the evaporation temperature, generating a vapor recoil force acting on the top of the molten pool, which makes the head of the molten pool sink, as shown in Figure 2b,h. The recess at the head of the molten pool forms a clear keyhole at 40 μs, and its bottom penetrates into the substrate, reaching a depth of 80 μm while still developing. The vapor recoil force and the Marangoni effect push the melt together to the tail of the molten pool, raising the surface of the tail. As the laser works, the depth of the keyhole continues to increase, as shown in Figure 2c,i, reaching about 110 μm below the powder bed at 160 μs and then tending to stabilize, as shown in Figure 2d,j. After the laser is turned off, the keyhole is gradually filled with the backflow melt, while leaving a solidified hole with a depth of 69.4 μm, forming a solidified track in a sloped shape, where the top surface gradually decreases from the tail to the head, as shown in Figure 2f,l. The animation of this simulation can be found in the Supplementary Materials, where the VS1 and VS2 show it in three-dimensional and longitudinal-section views, respectively.

**Figure 3:**
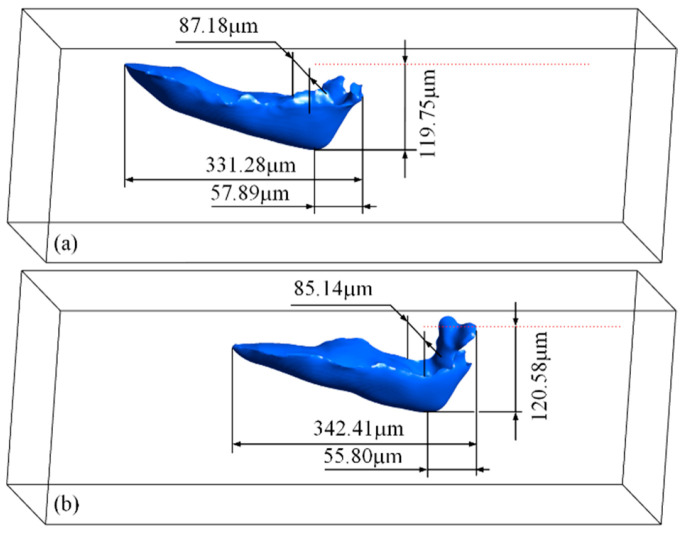
The profile indicated by the liquidus isothermal surface of the molten pool during the laser-scanning process. (**a**) t = 320 μs; (**b**) t = 480 μs.

The profile of the longitudinal section of the molten pool during the laser-scanning process is indicated by the solid blue line in Figure 2, and the three-dimensional morphology of the stable molten pool is shown in Figure 3. The molten pool presents a wedge shape. The depth of the molten pool gradually increases along the laser scanning direction, reaching the maximum depth near the laser spot of about 120 μm and a length of about 340 μm. The width is almost maintained at about 85 μm except slightly narrowed near the tail. Additionally, the horizontal distance between the front of the molten pool and the deepest point is relatively close, and the maximum is about 55 μm.

**Figure 4:**
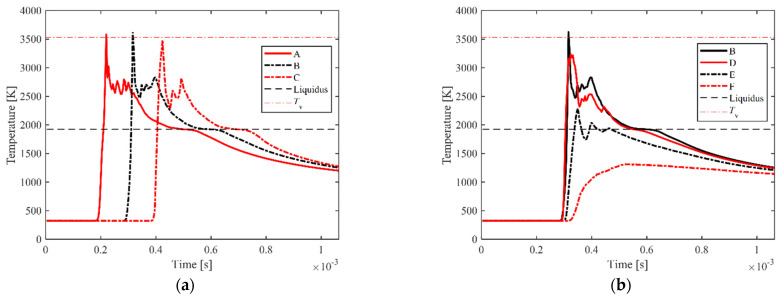
Temperature history of the characteristic points during the laser-scanning process, in which the positions of these points are shown in Figure 2f,l: (**a**) temperature history of the characteristic points along the scanning direction; (**b**) temperature history of the characteristic points vertical to the scanning direction.

The temperature evolution of the characteristic points in the track during the laser-scanning process (the position of these points is marked in Figure 2f,l is shown in Figure 4. When the laser spot passes through those points in the laser path, their temperatures rapidly rise above the liquidus temperature, as shown in Figure 4a, and keep rising and stay above the evaporating temperature for a very short time. Once the temperature exceeds the boiling point, vapor recoil occurs, which strongly drives the melt to flow downwards, forming a strong convective motion in the molten pool and enhancing the heat transfer. Meanwhile, the liquid surface of the molten pool fiercely shakes, and the temperature fluctuates greatly. As the laser passes over, the temperature at the characteristic points gradually drops to near the liquidus, and the melt begins to solidify. Due to the release of the phase-change enthalpy, the cooling rate of the melt is significantly reduced near the liquidus. The temperature history also tells that the liquid-phase lifetime of the materials in the laser path is about 300 μs under the simulated condition. The temperature evolution of the characteristic points perpendicular to the laser-scanning direction is shown in Figure 4b. The temperature of points B and D located within the laser spot rises almost simultaneously, while the peak temperature at point B is slightly higher due to point B being closer to the center of the laser spot, reaching above 3500 K. The temperature of point E close to the edge of the molten pool slightly exceeds the liquidus, and the temperature of point F rises slowly and is always below the liquidus, which is located outside the molten pool and heated mainly by conduction.

**Figure 5:**
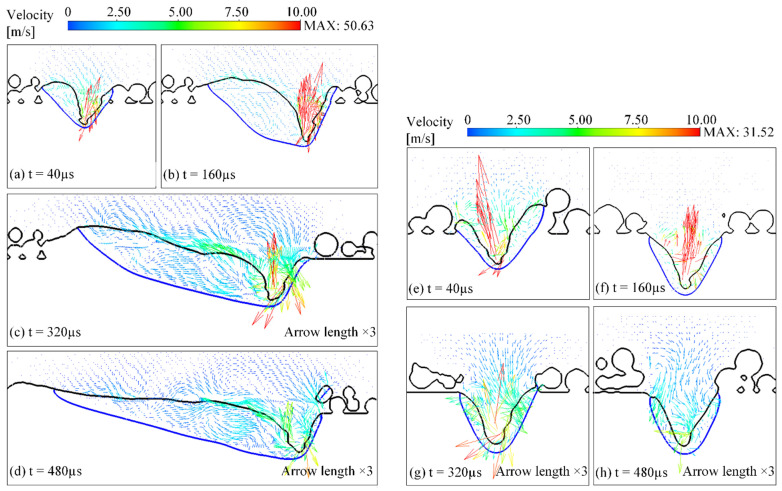
The velocity filed in (**a**–**d**) the longitudinal section and (**e**–**h**) the cross-section of the track during the laser scanning, where the blue line represents the boundary of the molten pool. To show the arrows of the velocity field more visibly, the length of the arrows in (**c**,**d**,**g**,**h**) is magnified by 3 times compared with those in the other subfigures (**a**,**b**,**e**,**f**).

Figure 5 shows the velocity field in the longitudinal section of the track as the laser is scanning the first layer. During the formation and development of the molten pool and the keyhole, the maximum velocity of the flow field appears on the side of the bottom of the keyhole, and the direction of movement is upwards, as shown in Figure 5a,b, up to about 50 m/s occasionally while about 10−15 m/s visibly. This indicates that the keyhole is subjected to a violent vapor recoil force, which pushes the surface of the molten pool to drop, forming a strong upward convection movement in the melt. When the depth of the keyhole tends to be stable, the maximum velocity also decreases to about 10 m/s, as shown in Figure 5c,d; the velocity of the melt away from the keyhole decreases to about 3 m/s and mostly below 1 m/s near the tail of the molten pool. As the keyhole moves forward steadily along the laser scanning direction, the melt flows backward in the skin layer of the molten pool under the combined action of the Marangoni effect and the vapor recoil force and then moves downward when approaching the boundary of the molten pool, forming a vortex in the flow field. Figure 5e−h shows the velocity field in the cross-section, which is similar to the velocity field in the longitudinal section.

**Figure 6:**
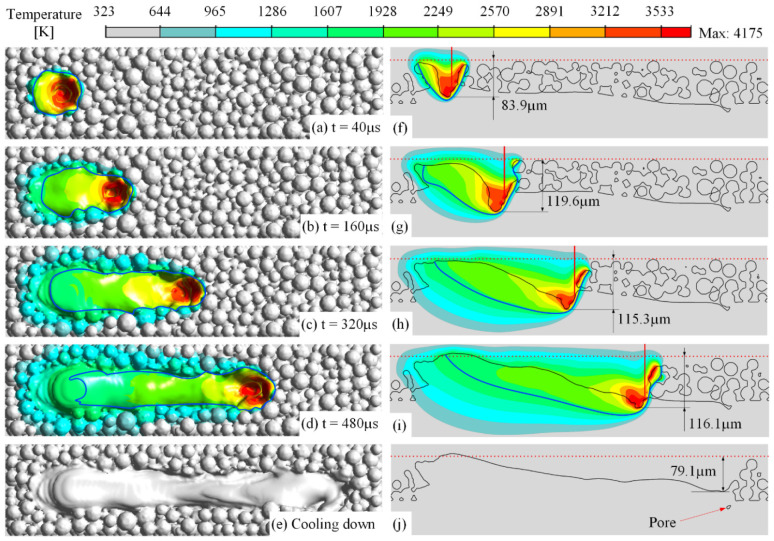
The evolutions of the track morphology and temperature field during the laser scanning on the second layer, where the laser power is 200 W, absorptivity coefficient 0.4, scanning speed 1 m/s, and layer thickness 50 μm. (**a**–**d**) the morphology of the track and the temperature field on its surface; (**f**–**i**) the same on the longitudinal section of the powder bed; (**e**,**j**) the morphology of the solidified track and the same on the longitudinal section.

To further demonstrate the application of the simulation framework, the powder-laying and laser-scanning processes of the second layer were simulated, as shown in Figure 6. Both the morphology of the molten pool and the temperature field distribution are similar to the first layer, but they also show some slight differences. Since the exact thickness of the second layer is usually thicker than the first layer due to the melt collapses, the heat transfer rate is slightly reduced, resulting in a slight increase in the depth of the keyhole, reaching about 117 μm. After fully cooling down, a solidified keyhole with a depth of 79.1 μm is left at the head of the solidified track, which is slightly increased compared with that in the first layer, and a pore defect is formed, as shown in Figure 6j. Additionally, under the stronger forces of the molten pool in the second layer, the left end of the top surface of the solidified track is bulged and slightly higher than the powder bed, resulting in a slight increase in the height difference between the head and the tail of the solidified track.

### 3.2. Effect of Laser Speed and Power

**Figure 7:**
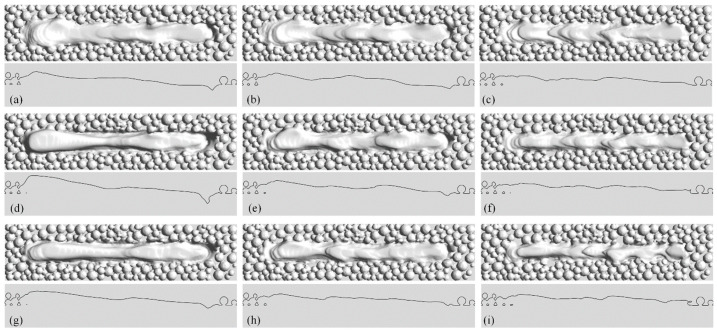
The morphology of the solidified track and the same on the longitudinal section under different laser powers and scanning speeds. (**a**) 200 W, 1 m/s; (**b**) 150 W, 1 m/s; (**c**) 100 W, 1 m/s; (**d**) 200 W, 1.5 m/s; (**e**) 150 W, 1.5 m/s; (**f**) 100 W, 1.5 m/s; (**g**) 200 W, 2 m/s; (**h**) 150 W, 2 m/s; (**i**) 100 W, 2 m/s.

To examine the effect of the laser power and scanning speed on the morphology of the molten pool, a series of simulations of a single track with laser power of 100, 150, and 200 W and scanning speed of 1.0, 1.5, and 2.0 m/s were carried out. The three-dimensional morphology of the solidified track and its profile in the longitudinal section are shown in Figure 7. The simulation results show that as the power increases, the solidification bulge at the beginning of the track increases while the depression at the end decreases. When the power is 150 W or 200 W, a residual keyhole appears at the end of the track, and the tip of the keyhole is lower than the top surface of the substrate. The height difference between the head and the tail of the track exceeds the thickness of the powder layer, as shown in Figure 7a,d,g. When the power is 100 W, the height difference is small, but more ripples appear on the top surface of the track, as shown in Figure 7c,f,i. As the power increases, the ripples gradually decrease, and the top surface of the track becomes smoother.

**Figure 8:**
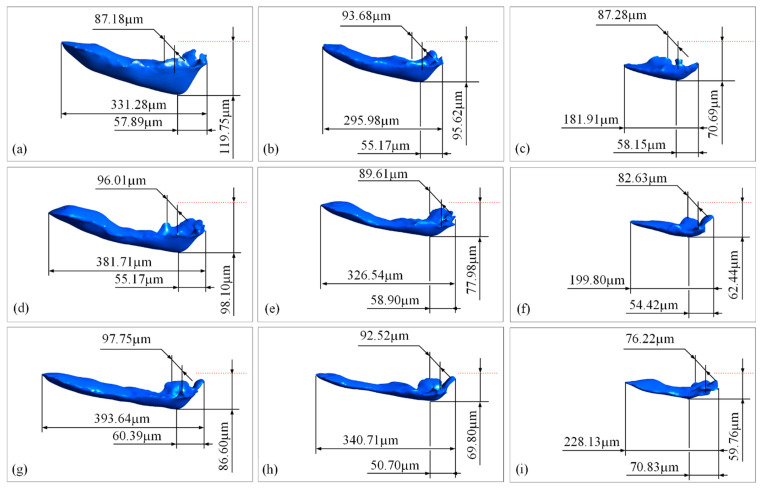
The profile of the molten pool, indicated by the liquidus isothermal surface during laser scanning under different laser powers and scanning speeds. (**a**) 200 W, 1 m/s; (**b**) 150 W, 1 m/s; (**c**) 100 W, 1 m/s; (**d**) 200 W, 1.5 m/s; (**e**) 150 W, 1.5 m/s; (**f**) 100 W, 1.5 m/s; (**g**) 200 W, 2 m/s; (**h**) 150 W, 2 m/s; (**i**) 100 W, 2 m/s.

Figure 8 shows the typical three-dimensional morphology of the molten pool obtained under different laser powers and scanning speeds. The results show that the laser process parameters have a significant impact on the size of the molten pool, but the morphology of the molten pool generally presents a wedge shape. The specific influences are as follows: (1) As the laser power increases, the length of the molten pool increases nonlinearly. From the comparison of the morphology of the molten pool under the same scanning speed but different laser powers, it can be seen that the length increases by about 100 μm as the power increases from 100 to 150 W, while the increment is only about 50 μm or less as the power increases from 150 to 200 W. (2) Under the same linear energy density of the laser power, the morphologies of the molten pools are quite different. Comparing the cases with 100 W, 1 m/s and 200 W, 2 m/s, the length of the molten pool obtained by the latter is significantly larger than the former and even more than doubled. (3) The scanning speed increases, the width of the molten pool slightly narrows, especially under a lower laser power of 100 W, and the length increases to a certain extent, but the increment ratio in the length is much smaller than that in the scanning speed. (4) As the laser power increases, the depth of the molten pool increases. When the laser power increases to 200 W and the scanning speed is 1 m/s, the increment in the depth of the molten pool is more significant than those at higher scanning speeds. This is mainly because there are more melts with a temperature exceeding the boiling point under this condition, resulting in a much stronger vapor recoil and then a deeper keyhole, which eventually leads to a significant increase in the depth of the molten pool.

**Figure 9:**
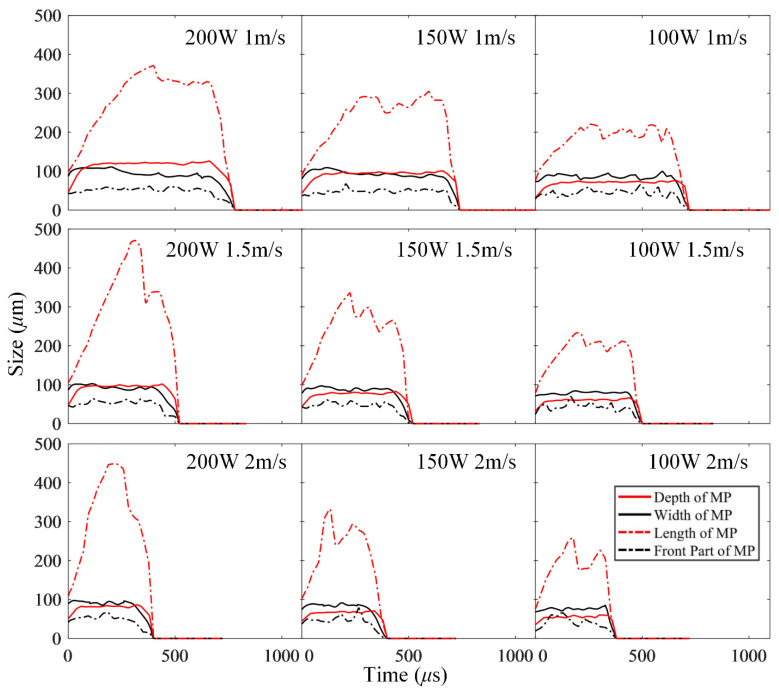
The dimensions of the molten pool (MP) during laser scanning under different laser powers and scanning speeds.

To study the evolution characteristics of the molten pool, the depth, width, length, and front distance of the molten pool under the aforementioned laser parameters were plotted in Figure 9. The results show the following: (1) When the laser power is low or the scanning speed is high, the depth of the molten pool is close to its front distance, about 60 μm, as the case of 100 W and 2 m/s shown in Figure 9. (2) As the power increases or the scanning speed decreases, the depth of the molten pool gradually approaches and even exceeds its width, reaching about 120 μm, as the case of 200 W and 1 m/s shown in Figure 9. (3) During the laser-scanning process, the stability of the depth of the molten pool is the highest, followed by the width and the front distance, and the length is the lowest, fluctuating in the range of tens of microns.

**Figure 10:**
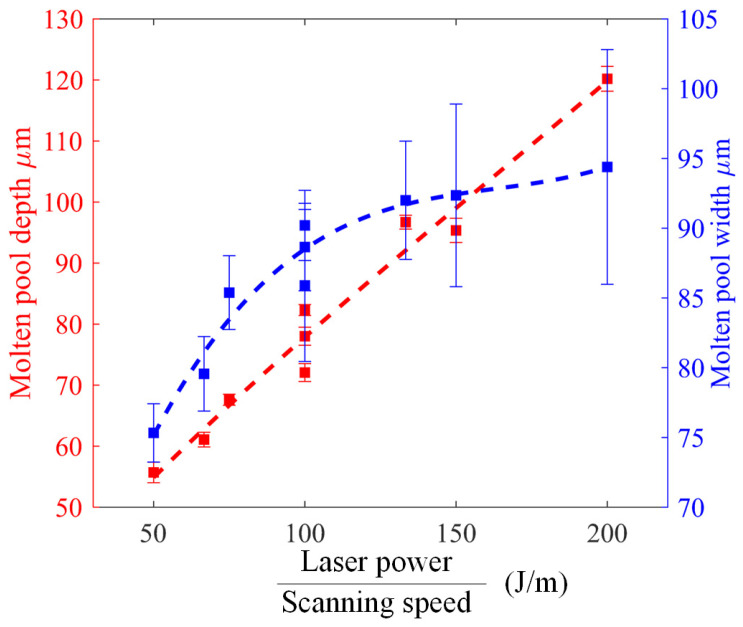
The dependences of the depth and width of the molten pool on the linear energy density of the laser power.

To further examine the dependence of the molten pool dimensions on the laser power and scanning speed, we plotted the depth and width of the molten pool versus the linear energy density of the laser power in Figure 10, where the linear energy density is the laser power divided by the scanning speed. The values of the depth and width were evaluated by averaging the molten pools when the melting is essentially stable, and the error bars indicate the size fluctuations. It can be seen that the depth increases almost linearly with the increase of the linear energy density while the width increases rapidly and then very slowly. Their increments indicate that the depth increases more than the width does. The error bars also indicate that the width of the molten pool shows much stronger fluctuation than the depth and the fluctuation is much stronger under a higher linear energy density of the laser power.

## 4. Conclusions

(1) This paper introduced a mesoscale DEM and CFD combined simulation framework for the simulation of the SLM process. The application of the framework showed a successful layer-by-layer simulation, which includes the alternate simulations of the power-laying and laser-scanning processes.
(2) The fundamental mesoscopic characteristics of the molten pool of the Ti-6Al-4V powder bed were found. The evolutions of the temperature history, flow field, keyhole, and the morphologies of the molten pool and the solidified track were presented and discussed in detail.
(3) The simulations by varying the laser power and scanning speed showed that as the laser power increases, the solidification bulge at the beginning of the track increases, the depression at its end decreases, the ripples on the top surface of the track gradually decrease and become smoother, and the length and width of the molten pool increase nonlinearly; as the scanning speed increases, the width of the molten pool slightly narrows, and the length increases to a certain extent, but the increment ratio falls far behind that of the scanning speed; there is a big difference in the morphology of the molten pool at the same linear energy density of the laser power; during the laser-scanning process, the stability of the depth of the molten pool is the best, the width and the front distance is the next, and the length is the worst.

It is well known that the response of the molten pool and keyhole to the process parameters are important to the SLM process control, and their data are also very useful to develop a more accurate part-scale model for additive manufacturing. The simulation framework presented in this work allows us to generate many credible simulation data, and then, we can collect and analyze these data using machine-learning methods, yielding a data-driven model, which would be helpful to achieve the aforementioned prospects. Furthermore, we know that there are other complex behaviors in the SLM process on the actual equipment, such as powder spattering. This sets a big challenge to couple the powder spattering and the molten pool dynamics appropriately, while we believe that it is possible to include the powder spattering in the molten-pool-concerned CFD model by coupling the DEM [38], alternatively adding an extra momentum source term, which can be derived from the volume expansion of the metallic vapor and surrounding gas to the spattering powders. This would need more effort to extend the presented mesoscopic model of the SLM process in the future.

## Figures and Tables

**Table 1:** Properties of Ti-6Al-4V and processing parameters.

Property/Parameter	Value	References
Density, *ρ*_m_/kg·m^−3^	4400	[27]
Surface tension coefficient, *γ*/N·m^−1^	1.53–0.28 × 10^−3^ (*T*-1941), *T* > *T*_sol_	[30]
Solidus, *T*_sol_/K	1877	[30]
Liquidus, *T*_liq_/K	1923	[30]
Evaporating temperature, *T*_v_/K	3533	[30]
Thermal conductivity, *k*_m_/W·m^−1^·K^−1^	$\left\{ \begin{array}{ll} 1.260+0.016T, & T\leq 1268\;K\\ 3.153+0.013T, & 1268<T\leq 1923\;K\\ -12.752+0.024T, & T>\;1923\;K \end{array}\right.$	[30]
Latent heat of melting, *L*_m_/J·kg^−1^	2.86 × 10^5^	[30]
Latent heat of vaporization, *L*_v_/J·kg^−1^	9.83 × 10^6^	[30]
Saturated vapor pressure at *T*_v_, *p*_0_/Pa	101,325	-
Specific heat capacity, *c*_p_/J·K^−1^·kg^−1^	$\left\{ \begin{array}{ll} 483.04+0.22T, & T\leq 1268\;K\\ 412.70+0.18T, & 1268<T\leq 1923\;K\\ 831.0, & T\;>1923\;K \end{array}\right.$	[30]
Radius of the laser spot, *ω*/μm	37.5	-
Scanning speed, *v*/m·s^−1^	1.0–2.0	-
Laser power, *P*/W	100–200	-
Absorptivity coefficient, *A*	0.4	[30]
Radiation coefficient, *ε*	0.4	[27]

## Supplementary Materials

The following supporting information can be downloaded at: https://www.mdpi.com/article/10.3390/ma15082850/s1, Video S1: Evolutions of the molten pool and the temperature during the laser-scanning process at 200 W and 1 m/s (Three-dimensional view); Video S2: Evolutions of the molten pool and the temperature during the laser-scanning process at 200 W and 1 m/s (Longitudinal-section view).
